# Face-to-Face Cognitive-Behavioral Therapy for Irritable Bowel Syndrome: The Effects on Gastrointestinal and Psychiatric Symptoms

**DOI:** 10.1155/2017/8915872

**Published:** 2017-01-22

**Authors:** Hanna Edebol-Carlman, Brjánn Ljótsson, Steven J. Linton, Katja Boersma, Martien Schrooten, Dirk Repsilber, Robert J. Brummer

**Affiliations:** ^1^Nutrition-Gut-Brain Interactions Research Centre, Örebro University, 701 82 Örebro, Sweden; ^2^Department of Clinical Neuroscience, Division of Psychiatry, Karolinska Institutet, Stockholm, Sweden; ^3^Center for Health and Medical Psychology (CHAMP), School of Law, Psychology and Social Work, Örebro University, Örebro, Sweden

## Abstract

Irritable bowel syndrome (IBS) is a gastrointestinal disorder linked to disturbances in the gut-brain axis. Visceral hypersensitivity and pain are hallmarks of IBS and linked to the physiological and psychological burden and to the nonadaptive coping with stress. Cognitive-behavioral therapy (CBT) for IBS has proven effective in reducing gastrointestinal and psychiatric symptoms in IBS by means of coping with stress. The present pilot study evaluated for the first time whether CBT for IBS affected visceral sensitivity and pain. Individual CBT was performed for 12 weeks in 18 subjects with IBS and evaluated in terms of visceral sensitivity and pain during rectal distensions using the barostat method and self-rated visceral sensitivity and gastrointestinal and psychiatric symptoms. Visceral discomfort, urge, and pain induced by the barostat were not affected by CBT but were stable across the study. However, the level of self-rated visceral sensitivity and gastrointestinal and psychiatric symptoms decreased after the intervention. Central working mechanisms and increased ability to cope with IBS-symptoms are suggested to play a key role in the alleviation of IBS symptoms produced by CBT.

## 1. Background

Irritable bowel syndrome (IBS) is a common multifactorial functional gastrointestinal disorder with a point prevalence of 11% in the adult western population [1]. Clinical symptoms include visceral hypersensitivity, abdominal pain, discomfort, altered gastrointestinal motility, and secretion as described in the Rome criteria [2]. Its pathophysiology is not fully understood but a multicomponent conceptual model involving physiological, affective, cognitive, and behavioral factors has been postulated [3]. Visceral hypersensitivity is a key hallmark of IBS that involves pain originating from the intestinal organs and is poorly understood in terms of its etiology and management or treatment. It is enhanced by stress, anticipation, and inflammatory factors as implicated in preclinical and clinical studies in the context of for example, gender, gut microbiota, immune functioning in IBS [4], and neonates maternally separated [5]. Visceral sensitivity can be pharmacologically and psychologically manipulated as was done in a study of hypnotherapy for IBS in which normalized levels of visceral sensitivity, GI, and psychological symptoms were reported [6]. Rectal thresholds can be adversely affected by acute and psychological stress and cognition influences pain perception shown by pain-rating scores higher during attention towards aversive stimuli than during distraction task in healthy volunteers [7]. Cognitions involving catastrophizing, rumination, and maladaptive coping are examples of cognitive-affective factors that play a role in the exacerbation of stress and IBS symptoms [8].

Visceral sensitivity is a robust hallmark of IBS. Previous studies, selecting the visceral stimuli of a barostat as a stressful trigger to assess visceral hypersensitivity in IBS, report increased attention via dorsal anterior cingulate cortex (ACC) towards pain sensation in IBS versus healthy controls [9], as well as impaired inhibitory control of rectal pain in emotional-sensory regulatory areas including amygdala, insula, and thalamus, as well as primary and secondary sensory cortex [10]. Acute tryptophan depletion in healthy volunteers during rectal stimuli to induce IBS-like processing of stressful pain altered hormonal response and decreased negative amygdaloid feedback to, for example, ACC, which yielded hypervigilance and amplified pain scores [11–13]. Autonomous nervous system reactivity during barostat assessment is also typically presented in IBS and provides further evidence that rectal hypersensitivity is not only a gastrointestinal symptom of IBS but also a reliable indicator of IBS-related cognitive impairment including abnormal processing of rectal pain and response to stress [14].

Cognitive-behavioral therapy (CBT) for IBS emphasizes cognitive, emotional, and behavioral strategies to better cope with physiological and psychological stressors. Previous studies report on the effectiveness of CBT for IBS in terms of improved gastrointestinal symptoms, quality of life, and the role of stress management as key mechanisms to regulate IBS pathophysiology [15–21]. The treatment, delivered over the Internet, showed promising results regarding gastrointestinal and psychological symptoms associated with IBS, as well as quality of life [15–21]. Our first study of CBT for IBS in a face-to-face format [22] reported improved gastrointestinal and psychological function, as well as improved quality of life in the majority of the subjects. However, there has not yet been a study on the actual visceral sensitivity and pain during an ecologically valid condition. Because we included measures of visceral sensitivity and pain during rectal stimulation in the study above (22) using the barostat method, this paper explores bidirectional gut-brain interactions by means of a top-down (i.e., brain initiated) intervention in subjects with IBS.

*Aim*. The aim of the present pilot study was to elucidate whether or not a cognitive-behavioral intervention for IBS affects (1) visceral sensitivity and pain during rectal stimuli using the barostat method in subjects with IBS and (2) self-rated visceral sensitivity and fear and worry about symptoms, as well as gastrointestinal symptoms in patients with IBS. Specifically, we asked whether face-to-face CBT for IBS affects

1. visceral pain, discomfort, and/or urge in subjects with IBS during rectal distensions at the pressure of 20, 30, 40, and 50 mmHg, respectively,
2. the continuous tolerable pressure (mmHg) at 20, 40, 60, and 80% of the estimated maximum of the highest visual analogue score, respectively, in subjects with IBS,
3. self-rated visceral sensitivity and gastrointestinal and psychiatric symptoms in subjects with IBS.

## 2. Methods

### 2.1. Design

The study included 18 subjects with IBS whose ratings of visceral pain, urge, and discomfort as well as the induced visceral pressure (mmHg) during rectal distensions with the barostat method were measured four weeks before the CBT intervention (t1, *n* = 8) and/or just before the CBT intervention (t2, *N* = 15) as well as after the intervention (t3, *N* = 15). In addition, subjects self-rated visceral sensitivity, symptoms of depression and anxiety, and gastrointestinal complaints of IBS were measured four weeks before (*n* = 13), just before (*N* = 18), and after the intervention (*N* = 18). Dependent variables were visceral sensitivity during rectal distensions (ratings of pain, urge, and discomfort using 100 mm VAS scales at the pressure of 20, 30, 40, and 50 mmHg), the continuous and maximal tolerable pressure (mmHg at 20, 40, 60, and 80% of the estimated maximum of the highest VAS score), and self-rated visceral sensitivity and gastrointestinal and psychiatric symptoms (visceral sensitivity index (VSI), gastrointestinal symptoms rating scale (GSRS), and Hospital Anxiety and Depression Scale (HADS)).

### 2.2. Subjects

A total of 18 subjects (14 females and 4 men; mean age = 35, SD = 13.31) suffering from IBS symptoms for one to five (*n* = 6) or more than five (*n* = 12) years prior to inclusion and having been diagnosed with constipation (*n* = 5), diarrhea (*n* = 9), and unspecified (*n* = 3) or mixed (*n* = 1) type IBS at a gastroenterological clinic according to Rome III criteria were included [23]. All subjects were eligible for an IBS diagnosis according to Rome III self-ratings at the time of inclusion; none had other gastrointestinal or psychiatric disorders, but chronic medical disorders included polycystic ovary syndrome (*n* = 1) and asthma (*n* = 1).

Current medications for IBS included loperamide and/or sterculia gum (*n* = 7) and alternative treatments included probiotics (at any time, *n* = 9; regular use, *n* = 6). Social status was either married (*n* = 3), shared household (*n* = 6), or single household (*n* = 9). Ten participants had completed high school and the rest had graduated from college. Three persons were on sick leave because of IBS.

Criteria for participation included (1) fulfilling Rome III diagnostic criteria for IBS and pain/discomfort frequency at least 2 days a week in the last 12 weeks, (2) having VAS score of global assessment of abdominal pain and discomfort equal to or >35 mm, (3) age being between 18 and 65 years, and (4) signing informed consent. Criteria for exclusion were (1) concurrent or recent treatment with drugs affecting intestinal function or mood, for example, antidepressants, (2) concurrent or recent (<2 weeks) use of nutritional supplements or herb products affecting intestinal function or mood (e.g., aloe vera, St. John's Wort), (3) depression or suicide tendencies according to Montgomery Åsberg Depression Rating Scale-Short (MADRS-S) screen [24] and/or clinical judgment, (4) abuse of alcohol or drugs according to Alcohol Use Disorder Identification Test screen [25] and/or clinical judgment, and (5) ongoing titration of psychopharmacological treatment.

### 2.3. The CBT Intervention

After a baseline, participants were introduced to their CBT therapist and the intervention was performed individually with 12 weekly one-hour sessions. During the whole study period, participants filled out weekly ratings about their gastrointestinal and psychological health using the online dedicated web portal [26]. Participants also filled out paper and pencil diaries consisting of five questions about their gut health using a five-point response scale [22].

Six clinical psychologists and two last-term psychology students at the Center for Health and Medical Psychology, CHAMP, at Örebro University, familiar with conducting CBT, were trained to conduct the CBT intervention. Supervision was provided by experienced psychologists (coauthors Brjánn Ljótsson and Steven J. Linton). Twelve participants performed a four-week baseline and six participants started treatment directly within a week from the first barostat assessment. The intervention was based on techniques and a manual developed and tested previously [18, 19]. The main modules of the manual consisted of exposure and mindfulness components. The total treatment entailed 12 sessions of therapy provided on an individual basis and guided by the manual. Thirteen participants participated in one session per week and the remaining five participants participated in the same therapy distributed over six weeks because of time constraints.

### 2.4. Rectal Pain Induction with the Barostat Method

To reduce the influence of a adipose tissue mass and abdominal wall tone during the barostat assessment, subjects fasted 12 hours prior to assessment and were placed in the left lateral decubitus position; the rectal probe was lubricated and placed 10–15 cm into the rectum. The probe consists of a 700 mL polyethylene bag secured on a rectal catheter (external diameter = 18 French). Rectal distensions were applied with a barostat (Electronic barostat, distender series II; G & J Electronic Inc., Toronto, Ontario, Canada) according to our previous study [11]. The barostat protocol consists of intermittent semirandom staircase distensions of 60 seconds' duration (15, 10, 25, and 20 mmHg, etc.) separated by an interval of 30 seconds of baseline pressure. The end point to stop the series of distensions is the perceptual threshold for maximal tolerable pain, discomfort, and/or urge or if the safety value of the maximal volume of 600 mL is exceeded. During each distension (after 13 seconds of distensions), subjects are asked to report their perception of pain, discomfort, and urge, respectively, using 100 mm VAS scales (no pain/discomfort/urge maximal tolerable pain/discomfort/urge). The barostat protocol has previously been approved by EPN (Etikprövningsnämnden, Ethical Review Board, Drn 2010-261, 2010-08-11, Dnr 2010-282, 2010-08-25) and described in detail [27].

### 2.5. Measurement of Symptoms with Rating Scales

#### 2.5.1. Gastrointestinal Symptoms Rating Scale (GSRS)

This is a 15-item clinical rating scale for gastrointestinal symptoms scored on a 7-point Likert scale in which 1 represents no symptoms and 7 the highest level of symptoms. The reliability and validity of the GSRS are well documented [28]. Interrater reliability is excellent and ranges from 0.86 to 0.90 for the separate items and from 0.92 to 0.94 for different gastrointestinal syndromes [28].

#### 2.5.2. Visceral Sensitivity Index (VSI)

This 15-item self-report questionnaire [29] measures gastrointestinal symptoms in IBS including unique aspects of anxiety related to gastrointestinal sensations, fear and hypervigilance that accompany misappraisals of GI-specific sensations, and discomfort. Items cover symptomatic areas like pain, diarrhea, constipation, bloating, or sense of urgency and are rated on 6-point scale ranging from strongly disagree (0) to strongly agree (5). VSI demonstrates excellent reliability (Cronbach's alpha = 0.93) as well as good content, convergent (0.61–0.66, *p* < 0.001), divergent, and predictive validity [29].

#### 2.5.3. Hospital Anxiety and Depression Scale (HADS)

This clinical scale [30] is a widely used and well-validated 14-item self-rating questionnaire that taps symptoms of depression and anxiety with seven questions, respectively. The instrument was originally developed to detect anxious and depressive symptoms in patients with physical health disorders. In a systematic overview of the HADS literature [31], correlations between the subscales varied from 0.40 to 0.74, Cronbach's alpha for the anxious subscale varied from 0.68 to 0.93, and the depressive subscale varied from 0.67 to 0.90. In most studies, an optimal balance between sensitivity and specificity (i.e., 0.80 for both) was achieved when the cut-off was set at ≥8 points on both subscales. HADS has been found to perform well in terms of evaluating and indicating cases of anxiety disorders and depression in somatic, psychiatric, and primary care patients [31].

### 2.6. Statistical Analyses

The slopes of a fitted linear function of the estimated VAS scores for pain, discomfort, and urge, respectively, at the fixed pressures of 20, 30, 40, 50, and 60 mmHg as well as the maximum tolerable pressure, that is, the highest visceral pressure that each subject could tolerate, were calculated and the fitted slopes were analyzed with paired samples *t*-tests (*ps* < 0.05).

The pressure (mmHg) of the barostat was measured but in order to achieve better comparability across subjects it was normalized based on 20, 40, 60, and 80% of the largest fitted VAS score using fits of logistic functions with intercept (see below). To characterize a subject's VAS score-pressure-curve, normalized pressures are reported for the above quantiles of the largest fitted VAS score value. The resulting normalized pressures were compared with paired samples *t*-tests (*ps* < 0.05) before and after CBT therapy. The function for the fitting of the observed scores was score = *K*/(1 + *e*^∧^(*r∗*(*x* − *d*))) − *K*/(1 + *e*^∧^(−*r∗d*)), with measured pressure *x* and parameters *K* (limit of the logistic function), *d* (shift with respect to pressure), and *r* (slope of the logistic function). The second term of the fitted function represents the fitted score at zero pressure, forcing a zero intercept for better comparability. The fit was obtained using the Levenberg-Marquardt algorithm, as implemented in R-package nlsLM [32, 33]. The formula for the pressure at 80%-of-max-VAS score estimate was *x*.80 = log(*K*/(0.8*∗*max.score + *K*/(1 + *e*^∧^(−*r∗d*))) − 1)/*r* + *d*, with “max.score” being the largest fitted VAS score value. Normalized pressures for 20, 40, and 60% of max-VAS scores were calculated accordingly.

The total score for the visceral sensitivity index (VSI), the Gastrointestinal Symptoms Ratings Scale (GSRS), and the Hospital Anxiety and Depression Scale (HADS) as well as the subscores for anxiety and depression on the HADS was analyzed with paired samples* t*-tests and for the skewed data according to the Shapiro-Wilk test of normality, the Wilcoxon signed ranks test was applied (*p* < 0.05). Because several *t*-tests were made, significance levels were adjusted with a Bonferroni correction. Effect sizes are reported in terms of Cohen's d for paired comparisons. In case of a significant difference between t1 and t2 (four weeks and directly before CBT; *n* = 12) for a certain dependent variable, as assessed with paired samples *t*-tests, the difference between t2 and t3 for that dependent variable was examined with an ANCOVA (*n* = 12), controlling for the (centered) difference between t1 and t2. The comparison of t2 and t3 was also assessed for all variables with paired samples* t*-tests and skewed data was analyzed with the nonparametric Wilcoxon signed rank test.

The statistical power was calculated post hoc using the g^*∗*^power software [34] based on a two-way paired samples *t*-test with an achieved effect size of around 0.48, an alpha level of 0.05, and a total sample size of 18. The power of the study was around 0.45, which is lower than previous studies on the efficacy of CBT for IBS [18, 35]. In order to achieve a power of 0.80 for the ANS measurement, a total sample size of at least 38 participants (SD = 1) would have been required which was not feasible for the present study.

### 2.7. Ethics

The study was approved by the ethical board EPN in Uppsala (Drn 2013/275) and conducted according to good clinical practice and the ethics of the Helsinki declaration [36].

## 3. Results

### 3.1. Sample Characteristics

Two of the original 20 subjects included in the study terminated their participation because of personal reasons and because of a lack of motivation for performing the exposure therapy, respectively. For the barostat analysis, data from three subjects could not be analyzed because of technical limitations during the barostat assessment; this left a total of 15 participants for the barostat analysis. A total of 12 participants at t1 and 18 participants at t2 and t3 were left for the analyses of the rating scales.

### 3.2. Discomfort, Pain, and Urge at the Pressure of 20, 30, 40, and 50 mmHg

**Figure 1:**
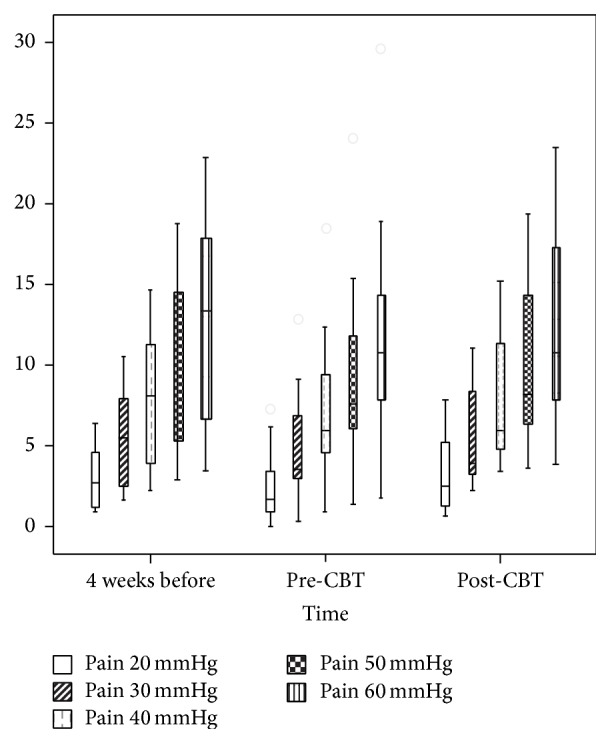
The estimated visceral pain based on ratings made by subjects with IBS at the rectal pressure of 20, 30, 40, 50, and 60 mmHg, respectively, four weeks before CBT, before, and after CBT. The pressure was induced by a barostat device.

Paired samples* t*-tests were conducted to compare the estimated ratings of discomfort, pain, and urge, respectively, at the pressure of 20, 30, 40, and 50 mmHg before and after CBT (t2-t3) as well as for the two rectal assessments performed before the intervention (t1-t2). None of the comparisons yielded significant results. For means and standard deviations, see Table 1. For a graphical presentation of the estimated visceral pain, see Figure 1.

### 3.3. Pressure at 20, 40, 60, and 80% of the Highest VAS Ratings

**Figure 2:**
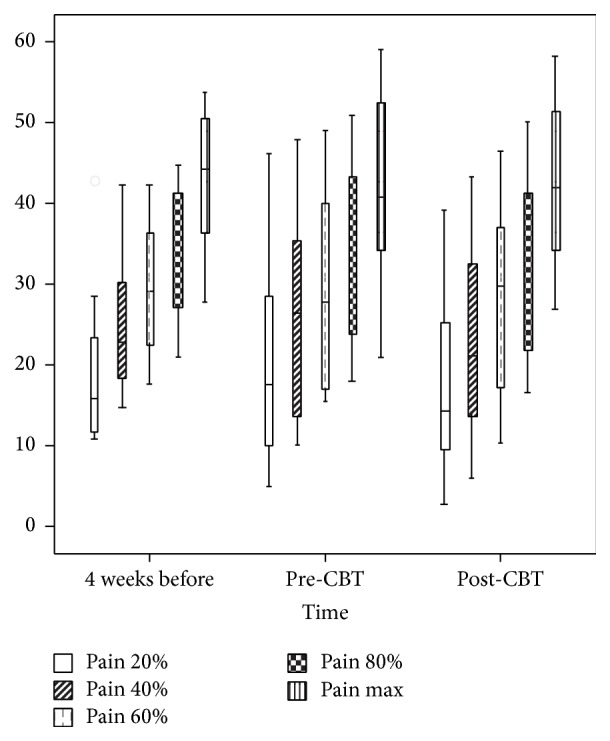
The actual pressure (mmHg) to the rectum at 20, 40, 60, 80, and 100% of the maximal tolerable rectal pain according to ratings made by subjects with IBS four weeks before CBT, before, and after CBT. The pressure was induced by a barostat device.

Paired* t*-tests were conducted to compare the estimated ratings of discomfort, pain, and urge, respectively, at 20, 40, 60, and 80% of the highest VAS rating before and after CBT (t2-t3) as well as for the two rectal assessments performed before the intervention (t1-t2). None of the comparisons yielded a significant difference. For means and standard deviations, see Table 2. For a graphical presentation of the pressure (mmHg) at 20, 40, 60, 80, and 100% of the maximal tolerable rectal pain, see Figure 2.

### 3.4. Maximal Tolerable Pressure

The participants' maximum tolerable pressures were calculated and compared before and after CBT (t2-t3). On average, participants maximum tolerable pressure did not improve after (M = 42.55, SD = 10.69) as compared to before (M = 42.05, SD = 11.76, *t*(14) = −0.174, *p* = 0.864) the intervention. A paired samples* t*-test was conducted for the maximal tolerable pressure for the two rectal assessments performed before the intervention (t1-t2). On average, participants' maximal tolerable pressure did not differ at t1 (M = 42.44, SD = 8.62) compared to t2 (M = 37.59, SD = 7.94, *t*(7) = 0.996, *p* = 0.352). Figure 2 presents a graphical presentation of the maximal tolerable rectal pain.

### 3.5. Self-Rated Symptoms

Participants visceral sensitivity decreased after as compared to before the intervention (*t*(17) = −5.980, *p* = 0.000) but did not differ at t1 compared to t2 (*t*(12) = 1.945, *p* = 0.076), although there was a trend towards lower scores at t2. Gastrointestinal symptoms decreased after as compared to before the intervention (*t*(16) = −3.606, *p* = 0.002) but did not differ at t1 compared to t2 (*t*(12) = 0.559, *p* = 0.587). The hospital anxiety and depression total score decreased after the intervention as compared to before the intervention (*t*(17) = −2.224, *p* = 0.040) but did not differ for t1 and t2 (*t*(11) = −1.00, *p* = 0.339). Anxiety subscores did not decrease after as compared to before the intervention (*t*(17) = 1.393, *p* = 0.181) and did not differ for t1 and t2 (*t*(11) = 0.312, *p* = 0.761). Depression subscores decreased after as compared to before the intervention (*t*(17) = −3.073, *p* = 0.007) but did not differ for t1 and t2 (*t*(11) = −1.688, *p* = 0.119). For means and standard deviations, see Table 3.

## 4. Discussion

The present pilot study investigated for the first time whether or not induced rectal pain and hypersensitivity can be modulated in IBS by means of CBT. The study also evaluated whether CBT affected self-rated visceral sensitivity and gastrointestinal and psychiatric symptoms in subjects with IBS. Our results show that visceral pain, discomfort, and urge during rectal distensions were not affected by the CBT treatment. Likewise, the continuous and the maximal tolerable rectal pressure were not affected by CBT. Moreover, there was no change in visceral sensitivity and rectal pain across the entire study. However, self-rated visceral sensitivity, gastrointestinal symptoms, and psychiatric symptoms of anxiety and depression did significantly decrease after CBT.

CBT for IBS did not seem to affect either the physiological perception or the intensity of visceral sensitivity and pain. The finding is consistent with earlier studies on IBS [37] suggesting high levels of stress and difficulties coping with anxiety and ANS activity in general and during physically and psychologically stressful situations in particular. Despite the fact that CBT improves gastrointestinal, visceral, and psychiatric symptoms according to the present and a previous study [22], it does not seem to affect the physiological perception and intensity of visceral pain during rectal distensions, which suggests more of central coping mechanisms related to IBS rather than physically altered functions regarding the disorder.

CBT seems to affect how participants cope with IBS, which in turn leads to reduced visceral sensitivity and gastrointestinal symptoms. In line with previous studies on CBT for IBS [22], the present study suggests that the intervention involves central mechanisms of coping rather than physiological visceral-afferent alterations of the gut. One plausible explanation of the CBT effect seen in the present study sample [22] and in other groups with IBS [15–21, 38] is suggested to be related to increased abilities to cope with IBS signs and symptoms which reduces manifestations of these symptoms [18, 19]. Several mediational analyses have also suggested that the effect of exposure-based CBT on IBS symptom is mediated through reduced symptom fear [20, 39, 40]. However, these meditational analyses have all been based on self-reported measures. The present study used for the first time the barostat technique to assess a biological marker of visceral pain and indicated that CBT effects arise primarily via psychological coping of IBS symptoms rather than visceral-afferent signaling of decreased symptoms.

The present study suggests that the effect of CBT upon IBS symptoms is initiated by improved psychological coping of IBS rather than an altered visceral-afferent physiological functioning, tolerance, and/or ability to perceive rectal pain, discomfort, urge, and pressure. In fact, the ability of the subjects to experience visceral pain and the intolerance of visceral pain seem to have remained constant, in contrast to the overall symptoms of IBS and associated impairments. Finally, improving IBS symptoms seem to be possible by means of altering psychological functioning related to the experience and coping of IBS symptoms, rather than altering IBS symptoms themselves.

### 4.1. Limitations

This study has some methodological confinements. First, post hoc calculations of the statistical power suggest only moderate power (0.45). Further, the effect sizes for many of the comparisons are limited to low to moderate effects. Taken together, these limitations reduce our ability to draw firm conclusions. Although we found a significant effect of CBT on IBS symptomatology in another report with these same participants, our sample size may be too small for physiological measures, for example, barostat assessment of visceral pain. Second, the lack of a control group raises questions as to whether the results of the present study were affected by psychological mechanisms not related to the intervention. Also, the selection of participants was not based on barostat data but only on self-ratings and clinical judgment, and thus the lack of a control group makes the presence of visceral hypersensitivity difficult to verify. Third, the evaluation took place immediately after finalizing the intervention and with no follow-up measurement and, thus, a more gradual effect on the intestinal visceral mechanisms may have been overlooked. Taken together, future research will need to test further the effects of CBT in randomized studies with larger samples.

## 5. Conclusions

The present study investigated for the first time whether CBT for IBS alters visceral sensitivity, pain, and the maximal tolerable pressure during rectal stimuli using the barostat method. While we did not find significant changes in the barostat measurements, the level of self-rated visceral sensitivity and gastrointestinal and psychiatric symptoms did decrease after the intervention. Changes in central working mechanisms and increased ability to cope with IBS symptoms are suggested to play a key role in the alleviation of IBS symptoms produced by CBT. More studies exploring the effect of CBT upon IBS in terms of biological markers and visceral pain during rectal distensions are needed.

## Figures and Tables

**(a):** 

mmHg	Pain, 4 weeks before		Pain, pre-CBT		Pain, post-CBT	
	M	SD	M	SD	M	SD
20	3.00	2.05	2.61	2.31	3.29	2.38
30	5.44	3.23	4.96	3.27	5.57	3.13
40	7.89	4.45	7.33	4.36	7.84	3.97
50	10.33	5.68	9.70	5.51	10.12	4.87

**(b):** 

mmHg	Discomfort, 4 weeks before		Discomfort, pre-CBT		Discomfort, post-CBT	
	M	SD	M	SD	M	SD
20	3.83	1.67	3.48	2.16	4.31	2.35
30	6.66	2.15	6.63	4.16	6.74	2.78
40	10.03	2.52	8.53	4.16	9.29	3.35
50	12.34	3.21	11.06	5.39	11.60	4.23

**(c):** 

mmHg	Urge, 4 weeks before		Urge, pre-CBT		Urge, post-CBT	
	M	SD	M	SD	M	SD
20	4.39	1.68	3.96	2.41	4.35	2.31
30	7.18	2.34	6.37	3.50	6.69	2.46
40	9.97	3.08	9.02	2.89	9.02	2.89
50	12.76	3.84	11.19	6.16	11.35	3.50

*Note*. Means (M) and standard deviations (SD) for dependent variables of visceral pain, discomfort, and urge in subjects with IBS during rectal distensions at the pressure of 20, 30, 40, and 50 mmHg collected four weeks before, just before (pre-CBT), and after CBT (post-CBT). *N* for the 4 weeks before measurement = 7; *N* for the pre- and post-CBT measurement = 14.

**(a):** 

%	Pain, 4 weeks before		Pain, pre-CBT		Pain, post-CBT	
	M	SD	M	SD	M	SD
20	19.40	10.77	20.54	11.86	17.69	10.18
40	24.95	9.42	25.32	11.86	23.39	11.07
60	28.08	11.57	29.49	8.86	28.08	11.57
80	34.23	8.75	33.46	11.55	28.70	12.08

**(b):** 

%	Discomfort, 4 weeks before		Discomfort, pre-CBT		Discomfort, post-CBT	
	M	SD	M	SD	M	SD
20	16.50	4.96	17.28	9.07	15.85	9.12
40	22.55	6.70	22.39	10.16	20.91	10.15
60	27.43	8.03	21.19	6.64	25.12	10.95
80	32.68	8.98	31.41	11.77	29.83	11.68

**(c):** 

%	Urge, 4 weeks before		Urge, pre-CBT		Urge, post-CBT	
	M	SD	M	SD	M	SD
20	13.39	3.22	13.78	6.96	14.65	8.62
40	19.54	5.02	19.03	9.12	19.69	10.03
60	23.37	11.02	24.74	6.85	23.91	11.11
80	30.51	8.47	28.12	12.86	28.70	12.08

*Note*. Means (M), and standard deviations (SD) for dependent variables, that is, the estimated pressure (mmHg) at 20, 40, 60, and 80% of the maximum ratings with regard to discomfort, pain, and urge, respectively, during the barostat assessment collected four weeks before (−4 weeks), just before (pre-CBT), and after CBT (12 weeks after CBT). *N* for the 4 weeks before measurement = 7; *N* for the pre- and post-CBT measurement = 15.

**Table 3:** Descriptive statistics for questionnaire data.

	−4 w			Day 0			Week 12		
	M	SD	95% CI	M	SD	95% CI	M	SD	95% CI
VSI	43.92	14.14	38.25, 53.91	41.06	17.21	31.35, 47.32	21.28^*∗∗*^	14.74	14.42, 26.41
GSRS	40.23	12.98	34.50, 49.50	37.83	14.67	31.04, 50.30	29.82^*∗∗*^	13.82	22.52, 41.32
HADS-T	14.00	5.66	10.41, 17.59	14.22	5.75	11.34, 18.16	11.39^*∗*^	6.39	7.96, 15.54
HADS-D	3.83	2.79	2.06, 5.61	4.5	2.81	2.91, 6.59	2.83^*∗∗*^	2.55	1.33, 4.67
HADS-A	10.17	3.30	8.07, 12.26	9.72	3.68	7.85, 12.15	8.56	4.19	6.36, 11.14

*Note*. Means (M), medians (Mdn), standard deviations (SD), and 95% confidence intervals (CI) for dependent variables of visceral sensitivity index (VSI), Gastrointestinal Symptoms Rating Scale (GSRS), and hospital anxiety and depression symptom scale (total, anxiety, and depression) in subjects with IBS collected four weeks before (−4 weeks), just before (day 0), and after cognitive behavioral therapy (CBT) (12 weeks).  ^*∗*^*p* < 0.05,  ^*∗∗*^*p* < 0.01.

## Abbreviations

ACC: Anterior cingulate cortex
ANS: Autonomous nervous system
CBT: Cognitive-behavioral therapy
GI: Gastrointestinal
GSRS: Gastrointestinal Symptoms Ratings Scale
HADS: Hospital Anxiety and Depression Scale
IBS: Irritable bowel syndrome
VAS: Visual Analogue Scale
VSI: Visceral sensitivity index.
